# Intrahepatic Infiltrating NK and CD8 T Cells Cause Liver Cell Death in Different Phases of Dengue Virus Infection

**DOI:** 10.1371/journal.pone.0046292

**Published:** 2012-09-26

**Authors:** Jui-Min Sung, Chien-Kuo Lee, Betty A. Wu-Hsieh

**Affiliations:** Graduate Institute of Immunology, National Taiwan University College of Medicine**,** Taipei, Taiwan, Republic of China; University of Rochester, United States of America

## Abstract

Elevated liver enzyme level is an outstanding feature in patients with dengue. However, the pathogenic mechanism of liver injury has not been clearly demonstrated. In this study, employing a mouse model we aimed to investigate the immunopathogenic mechanism of dengue liver injury. Immunocompetent C57BL/6 mice were infected intravenously with dengue virus strain 16681. Infected mice had transient viremia, detectable viral capsid gene and cleaved caspase 3 in the liver. In the mean time, NK cell and T cell infiltrations peaked at days 1 and 5, respectively. Neutralizing CXCL10 or depletion of Asialo GM1^+^ cells reduced cleaved caspase 3 and TUNEL^+^ cells in the liver at day 1 after infection. CD8^+^ T cells infiltrated into the liver at later time point and at which time intrahepatic leukocytes (IHL) exhibited cytotoxicity against DENV-infected targets. Cleaved caspase 3 and TUNEL^+^ cells were diminished in mice with TCRβ deficiency and in those depleted of CD8^+^ T cells, respectively, at day 5 after infection. Moreover, intrahepatic CD8^+^ T cells were like their splenic counterparts recognized DENV NS4B_99–107_ peptide. Together, these results show that infiltrating NK and CD8^+^ T cells cause liver cell death. While NK cells were responsible for cell death at early time point of infection, CD8^+^ T cells were for later. CD8^+^ T cells that recognize NS4B_99–107_ constitute at least one of the major intrahepatic cytotoxic CD8^+^ T cell populations.

## Introduction

Dengue virus (DENV) infection causes dengue fever (DF) which may progress to become life-threatening dengue hemorrhagic fever (DHF) and/or dengue shock syndrome (DSS). About 50 million cases of dengue infection are reported worldwide each year and dengue has become the most important tropical disease second to malaria [1].

The involvement of liver in DENV infection has been well documented. An early report showed that among 270 dengue cases studied, as high as 93% of them had elevated liver enzyme levels [2]. In most cases, the elevation is mild to moderate, indicating liver damage is not severe [2], [3]. Nevertheless, the level of aminotransfereases correlates with the severity of vascular leakage and bleeding manifestations [4], [5]. DENV infects primary human hepatocytes and Kupffer cells as well as hepatoma cell lines [6]–[9]. Hepatocytes and Kupffer cells as DENV targets are confirmed in biopsies and autopsies of fatal cases [3], [10]–[12]. Both apoptotic and necrotic cell death are detected in infected liver [11]. Even though DENV is demonstrated to cause HepG2 and Huh7 apoptosis [8], whether liver pathology and cell death is directly caused by viral infection remains unclear.

Immune activation has been proposed as a cause for severe dengue illness. The expression of CD69 on CD8^+^ T and NK cells is high in both DF and DHF, but higher in DHF patients [13]. Serum levels of soluble IL-2 receptor (sIL-2R), sCD4, IL-2 and IFNγ are higher in DF and DHF patients than in healthy donors, and sCD8 is high in only DHF but not DF patients, showing that CD8^+^ T cell activation correlates with severe disease [14]. Moreover, inflammatory cell infiltration is evident in liver biopsy of patients with high aminotransferase levels [10]. These studies suggest that immune cell activation and possibly intrahepatic infiltration may be involved in liver pathology.

Small animal models were developed to study DENV infection. Severe combined immunodeficient (SCID) mice xenografted with human cell lines showed viremia [15]–[17]. RAG2^−/−^γc^−/−^ mice engrafted with human CD34^+^ hematopoietic stem cells exhibited viremia and detectable anti-dengue immunoglobulins [18]. Injection of AG129 mice with low dose of non-mouse adapted DENV resulted in spleen damage, liver dysfunction and increased vascular permeability and eventual death [19]. BALB/c and C57BL/6 mice infected with DENV showed elevated liver enzymes and intrahepatic cellular infiltration [20]–[22]. The elevation of liver enzymes coincided with the kinetics of CD44^high^ mononuclear cell infiltration [21]. Together, these reports show that intrahepatic infiltration of activated immune cells positively correlates with DENV-induced liver damage. However, the direct proof of the relationship between them needs further investigation.

In this study, we employed immunocompetent mouse model to study the immunopathogenesis of DENV-induced liver injury. Intravenously infected mice experienced transient viremia and viral capsid gene was detected in the liver. Intrahepatic NK cell infiltration peaked at day 1 when TUNEL^+^ cells and cleaved caspase 3 appeared. Blocking or depleting NK cells diminished liver cell death. Cytotoxicity assay demonstrated that intrahepatic leukocytes were cytotoxic against dengue virus-infected Hepa 1–6 targets and CD8^+^ T cell depletion decreased liver cell death. Interestingly, intrahaptic CD8^+^ T cells, like splenic CD8^+^ T cells, recognized NS4B_99–107_. Taken together, we demonstrated for the first time in mouse model that intrahepatic infiltrating NK and CD8^+^ T cells cause liver cell death at different phases of infection respectively and that intrahepatic cytotoxic CD8^+^ T cells recognized NS4B_99–107_.

## Materials and Methods

### Mice

Wild type and TCRβ knockout (KO) mice in C56BL/6 background were obtained from the Jackson Laboratory (Bar Harbor, ME) and bred in the Laboratory Animal Center, National Taiwan University College of Medicine. STAT1 KO [23] were maintained in LEVEL Biotechnology (Taipei, Taiwan). This study was carried out in strict accordance with the recommendations in the Guidebook for the Care and Use of Laboratory Animals, The Third Edition, 2007, published by The Chinese-Taipei Society of Laboratory Animal Sciences. The experimental protocol was approved by the Committee on the Ethics of Animal Experiments of the National Taiwan University College of Medicine (Permit Number: 20080169).

### Virus and Infection

DENV-2 strain 16681 was propagated in C6/36 cells (ATCC CRL-1660, gift from Dr. Chuan-Liang Kao, Collage of Medicine National Taiwan University, Taipei, Taiwan). Viral titers were determined by plaque-forming assay on BHK cells. To obtain UV-inactivated virus, viral stocks were treated with UV at 50 mJ/cm^2^ in 30 sec by UVlink crosslinker (UVItec, UK). Mice were inoculated intravenously with 1×10^8^ PFU of viable virus or the equivalent UV-inactivated virus. To obtain higher numbers of intrahepatic leukocytes for cytotoxicity assay, a second injection of virus at day 7 after primary infection was given. STAT1 KO mice were inoculated intravenously with 1×10^7^ PFU of viable virus or the equivalent UV-inactivated virus. At indicated time points, mouse livers were perfused with PBS through the portal vein before harvest.

### RT-PCR and Real-time PCR

Serum RNAs were extracted by QIAamp Viral RNA Mini Kit (Qiagen, Hilden, Germany). RNAs in the liver and C6/36 cells cultured in medium containing mouse sera (at 1∶2 dilution) [20] were extracted by Trizol (Invitrogens, Grand Island, NY). RNAs were reversely transcribed by C14A (5′-AAT ATG CTG AAA CGC GAG AGA AAC CGC G-3′) and C69B (5′-CCC ATC TCT TCA GTA TCC CTG CTG TTG G-3′) [24] for viral capsid gene expression and by random primers (Protech, Taipei, Taiwan) for *β-actin*, *GAPDH* and chemokine gene expression. Viral capsid gene expression in C6/36 cells and liver tissues was then determined by PCR using primer pairs mouse *GAPDH*: forward 5′- ACC ACA GTC CAT GCC ATC AC -3′ and reverse 5′- TCC ACC ACC CTG TTG CTG T-3′ [25] C6/36 *β-actin*: forward 5′-CCA CCA TGT ACC CAG GAA TC-3′ and reverse 5′-CAC CGA TCC AGA CGG AGT AT-3′ [26]. Viral capsid gene in serum was determined by real-time PCR by MyiQ Single Color Real-Time PCR Detection System (Bio-Rad, Hercules, CA) using primer pairs: C16A: 5′-GCT GAA ACG CGA GAG AAA CC-3′ and C46B: 5′-CAG TTT TAG TGG TCC TCG TCC-3′ [27] and.gene expressions of *CCL5* and *CXCL10* in liver tissues as well as Hepa 1–6 cells were determined using primer pairs: *CXCL10*: forward 5′- GCC GTC ATT TTC TGC CTC A-3′ and reverse 5′- CGT CCT TGC GAG AGG GAT C-3′; *CCL5*: forward 5′- CAA GTG CTC CAA TCT TGC AGT C-3′ and reverse 5′- TTC TCT GGG TTG GCA CAC AC-3′ and *GAPDH*: forward 5′- GGC AAA TTC AAC GGC ACA GT -3′ and reverse 5′- AGA TGG TGA TGG GCT TCC C-3′ [28] were used. The relative levels of chemokine mRNA expression were normalized against *GAPDH* expression.

### ELISA

CXCL10 and CCL5 levels in serum were determined using ELISA antibody pairs purchased from Bender MedSystems (La Jolla, CA) for CXCL10 and R&D systems (Minneapolis, MN) for CCL5.

### Western Blotting

Protein was extracted from perfused liver tissues by RIPA lysis buffer containing 1% protease inhibitor mixture (Sigma-Aldrich), and then boiled in SDS sampling buffer, separated in 12% SDS-polyacrylamide gels, transferred to nitrocellulose membrane (GE Healthcare Bio-Sciences, Sweden) and blotted with anti-caspase 3 or anti-actin antibody (each at 1∶1000, Cell Signaling, Danvers, MA) followed by HRP-conjugated goat anti-rabbit IgG antibody (1∶2000, Jackson ImmunResearch Laboratories, West Grove, PA). The blots were visualized using the ECL detection system (Thermo, Rockford, IL). Actin was used as loading control.

### Isolation of Intrahepatic Leukocytes (IHLs)

After perfusion, the liver was incubated in digestion buffer (0.1% collagenase IV and 0.01% DNase I (Sigma-Aldrich)) at 37°C for 40 min. The digested tissues were pressed through 70 µm mesh (BD Falcon, Franklin Lakes, NJ) and the passed-through cells were collected and separated in Ficoll-Paque PLUS (GE Healthcare Bio-Sciences) by centrifugation at 2000 rpm (760 g) for 20 min. The intrahepatic leukocytes at the interface were collected.

### Immunohistochemistry and TUNEL Staining

Liver tissues were embedded in O.C.T. embedding medium (Shandon, CRYOTOME SME, Pittsburgh, PA), snap frozen in liquid nitrogen. Frozen tissues were cryosectioned at 5 µm-thicknesses. After fixation in acetone, rat anti-mouse CD49b (DX5) antibody (BD Pharmingen, San Diego, CA) was added and left at 4°C overnight. HRP-conjugated goat anti-rat Ig antibody (Jackson ImmunResearch Laboratories) was added and incubated at 37°C for 5 h. DAB (2,4-Diaminobutyric Acid, Sigma-Aldrich) was used as substrate for color development and hematoxylin was used as counterstain.

To detect dead cells, cryosectioned tissues were fixed in 4% paraformadelyde before treatment with 0.1% Triton X-100 on ice. TUNEL reaction mixture (Roche Applied Science, Indianapolis, IN) was added and left at room temperature for 60 min. Converter-POD (anti-FITC, Roche Applied Science) was then added and the slides were incubated at room temperature for another 30 min. DNaseI (1 µg/µl) treated sections were used as positive control. DAB substrate was added for color development. The slide was counterstained by methyl green.

### Antibody Preparation and Treatment

Hybridomas that produce anti-CD4 (GK1.5, ATCC TIB-207), anti-CD8, (2.43, ATCC TIB-210) and anti-CXCL10 (kindly provided by Dr. Thomas E. Lane, Molecular Biology and Biochemistry Research, University of Cailfornia, Irvine) antibodies were cultured in DMEM (Gibco BRL, Gaithersburg, MD) containing 10% FBS (Biological Industries, Kibbutz Beit Haemek, Israel). Supernatants were purified by HiTrap protein G column (GE Healthcare Bio-Sciences) and concentrated using Millipore Amicon (10,000 kD, Beverly, MA). Anti-CXCL10 antibody (250 µg) was injected intraperitoneally at one day before infection. GK1.5 and 2.43 (250 µg) were given intraperitoneally starting at the day of infection and every other day thereafter until termination of the experiment. Flow cytometry revealed that less than 1% of CD4^+^ and CD8^+^ cells were detected in the spleen and liver of anti-CD4 and anti-CD8 antibody treated mice, respectively. Polyclonal rabbit anti-mouse/rat Asialo GM1 (AGM1) antibody (37 mg/ml, Cedarlane, Ontario, Canada) was given at different volumes intravenously at one day before infection to deplete NK cells. Depletion of NK cells, CD4 or CD8 T cells did not increase virus capsid gene expression.

### Detection of Viral Antigen, Cell Surface Marker and Intracellular Cytokine

C6/36 cells were cultured in sera (at 1∶2 dilution) for 3 days before harvest and viral protein expression was determined by staining with rabbit anti-DENV antiserum (gift from Dr. Wen Chang, Academia Sinica, Taipei, Taiwan) [21] followed by PE-goat anti-rabbit antibody. Splenocytes and IHLs were collected at different time points after infection. Cells were stained with anti-CD4 (GK1.5), anti-CD8 (53-6.7) (eBioscience, La Jolla, CA) and anti-CD49b (DX5) (BD Pharmingen) antibodies. For intracellular staining, cells were fixed and then stained with anti-IFNγ antibody (eBioscience) in Perm/Wash buffer (containing 0.5% saponin). Cells were acquired by FACSCalibur and data were analyzed by CellQuest (BD Biosciences, San Jose, CA).

### Cytotoxicity Assay

Hepa 1–6 (H-2^b^) cells (ATCC CRL-1830) were infected with DENV at M.O.I. of 100 for 24 hours. Flow cytometry analysis showed about 50% of Hepa 1–6 cells were DENV-positive and 100% of cells were viable after 24 h of infection. IHLs harvested from mice at 3 days after given second injection of DENV were co-cultured with Hepa 1–6 in round bottomed plates at ratios of 1∶1, 5∶1 and 10∶1 for 5 h. CytoTox 96 non-radioactive cytotoxicity assay (Promega, Madison, WI) which determines the LDH release in culture supernatant was used to quantify cell lysis. Each datum point is the mean of triplicate determinations. The cytotoxicity percentage was calculated as the ratio of LDH release in experimental group to the total cell lysis group as manufacture’s instruction.

### Peptide Stimulation

Splenocytes or IHLs were harvested from infected wild type or STAT1 KO mice at different time points after infection. Peptides C_51–19_: VAFLRFLTI; E_451–458_: VSWTMKIL; NS2A_8–15_: FSLGVLGM; NS4B_59–66_: SSVNVSLT; NS4B_99–107_: YSAVNPITL; and NS5_237–245_: RMLINRFTM [29] were synthesized by GlycoNex (Taipei, Taiwan) and the purity was >95% as determined by HPLC. One million cells were stimulated with each individual peptide (0.1 µg/ml) in the presence of monensin (2 µM, Sigma-Aldrich) and soluble anti-CD28 antibody (1 µg/ml) for 6 h. IFN-γ-producing CD8^+^ T cells were determined by intracellular cytokine staining as described above.

### Statistical Analysis

Two-tailed Student *t* test was used to determine statistical significance.

## Results

### Animal Model to Study DENV-induced Liver Injury

**Figure 1:**
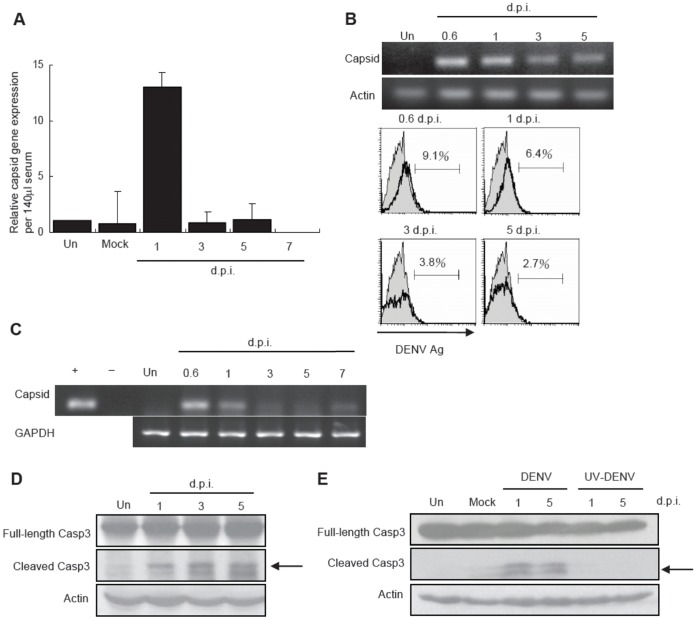
DENV administered intravenously causes transient viremia, liver cell infection and apoptosis. Viral capsid gene expression in the serum of uninfected mice (Un) or mice at different time points after infection of DENV (d.p.i., days after infection) was determined by RT real-time PCR (*n* = 2 per time point in each experiment). (B) C6/36 cells were cultured in medium containing serum collected from uninfected or infected mice. Viral capsid gene expression was determined by RT-PCR. Beta-actin of C6/36 cells was used as internal control (top). Viral antigen expression was determined by rabbit anti-DENV antibody staining (bottom). Shaded histograms outlined by thin line and open histogram outlined by thick line represent cells cultured in serum from uninfected and infected mice respectively. Data presented are representative of two independent experiments (*n* = 3 per time point in each experiment). (C) Liver was collected from uninfected (Un) and infected mice. Viral capsid gene expressions were determined by RT-PCR. Data presented are representative of two independent experiments (*n = *5 per time point in each experiment). (D and E) Liver was collected from uninfected (Un), mice infected with DENV and mice inoculated with otherwise equivalent UV-inactivated DENV (UV-DENV). Arrow points to cleaved caspase 3. Data presented are representative of five independent experiments (n = 3 per time point in each experiment).

Wild type mice intravenously infected with DENV strain 16681 have elevated serum levels of ALT and AST [21]. Here, we employed this model to investigate the mechanism of liver injury in DENV infection. RT-real-time PCR (Fig. 1A) and PCR (Fig. 1B) results show that dengue virus capsid gene was detectable in sera of infected mice at days 0.6 and 1 and viable virus detectable at days 0.6, 1, 3, 5 after infection. Capsid gene expression was also detected in liver tissues at the early phase after infection (Fig. 1C). While UV-inactivated DENV did not have any effect, transient DENV infection induced caspase 3 cleavage in the liver at days 1, 3 and 5 (Fig. 1D and E), showing that liver cells undergo apoptosis after infection. These data together demonstrated that accompanied elevated serum levels of ALT and AST [21], transient DENV infection induced liver cell apoptosis.

### Upregulation of CXCL10 and CCL5 after DENV Infection

**Figure 2:**
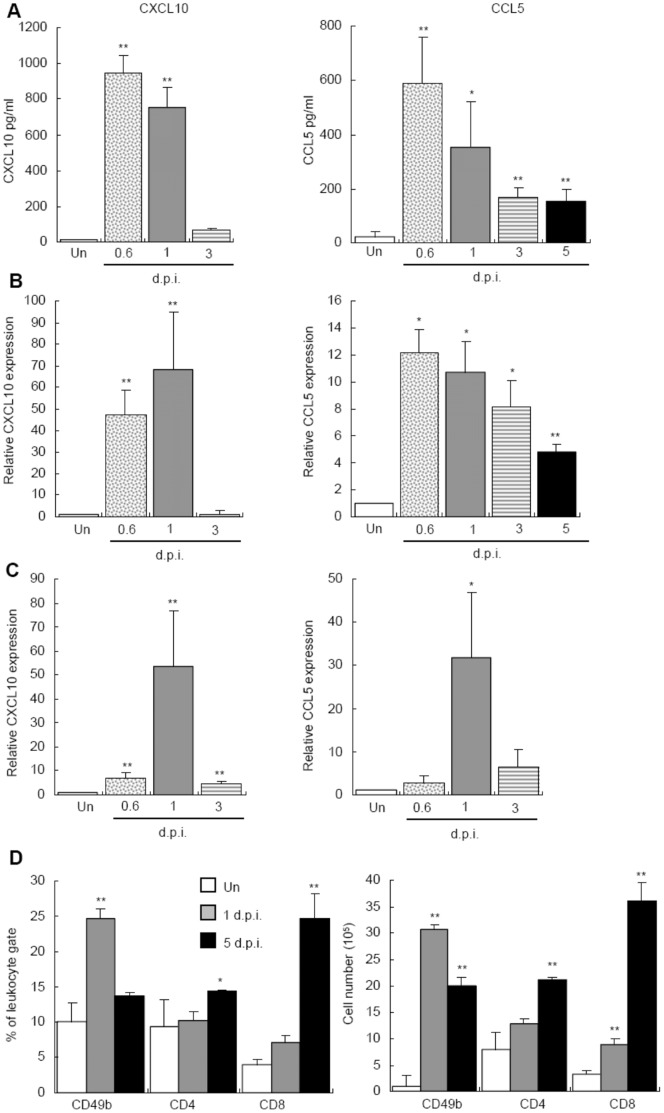
Intrahepatic infiltration of NK and T cells coincides with CXCL10 and CCL5 productions in the liver. (A) The levels of CXCL10 and CCL5 in sera collected from uninfected (Un) and infected mice were determined by ELISA. Data presented are from three independent experiments (*n = *3 per time point in each experiment). (B) *CXCL10* and *CCL5* mRNAs in the liver were analyzed by RT-real-time PCR and normalized against *GAPDH*. Data presented are from three independent experiments (*n = *3 per time point in each experiment). (C) Hepa 1–6 cells were infected with DENV at MOI of 1 and *CXCL10* and *CCL5* mRNA expressions were analyzed by RT-real-time PCR and normalized against *GAPDH*. Data presented are from three independent experiments (*n = *2 per time point in each experiment). (D) IHLs were collected from uninfected (open) or infected mice at days 1 (shaded bar) and 5 (darkened) after infection. The percentage in total IHL population (left) and the absolute number (right) of CD3^-^CD49b^+^ NK cells, CD3^+^CD8^+^ and CD3^+^CD4^+^ T cells were analyzed by flow cytometry (*n = *5 per time point in each experiment). * *P* value <0.05, ** *P* value <0.01 compared with uninfected groups.

Examining chemokine expression, we found that the protein and mRNA levels of CXCL10 were significantly elevated in the serum and liver of infected mice at days 0.6 and 1 after infection. The CCL5 levels were the highest at day 0.6 and remained significantly higher than controls until day 5 (Fig. 2A and B). In addition, DENV infection of mouse hepatoma cell line, Hepa1–6, induced the expressions of CXCL10 and CCL5 mRNA (Fig. 2C). These results indicate that CXCL10 and CCL5 are upregulated in the liver after DENV infection and hepatocytes are likely one of their source.

Analysis of intrahepatic leukocytes (IHL) revealed two waves of cellular infiltration into the liver after infection. While NK cells peaked at day 1, CD4^+^ and CD8^+^ T cells did at day 5 (Fig. 2D). Notably, intrahepatic CD4^+^/CD8^+^ T cell ratios changed from 1.4 in uninfected controls to 0.6 in infected mice at day 5. These results demonstrate that correlating to hepatic CXCL10 and CCL5 expressions, there is a transient intrahepatic infiltration of NK at day 1 and T cells at day 5 after DENV infection.

### NK Cell Infiltration Results in Liver Cell Death in Early Phase of Infection

**Figure 3:**
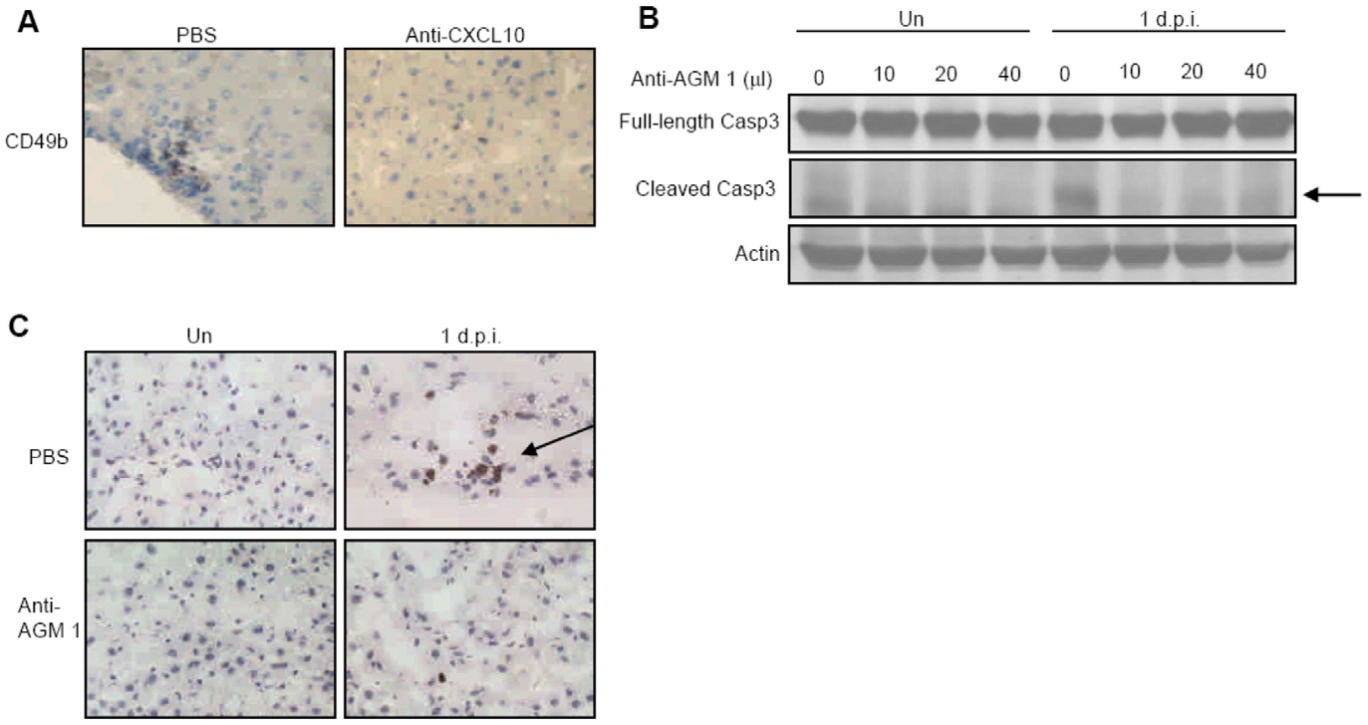
Intrahepatic infiltrating NK cells cause liver cell death at day 1 after DENV infection. (A) Liver cryosections collected from mice with or without anti-CXCL10 antibody at day 1 after DENV infection were stained with anti-CD49b antibody. Data are representative of three independent experiments (*n = *3 per time point in each experiment). (B) Uninfected and infected mice at day 1 were treated with different volumes of anti-AGM1 antibody. Arrow points to cleaved capase 3. Data presented are representative of four independent experiments (*n = *3 per time point in each experiment). (C) Liver cryosections collected from uninfected or infected mice with or without anti-AGM1 were stained with TUNEL reagents. Data are representative of three independent experiments (*n = *3 per time point in each experiment).

Figure 3A shows that neutralization of CXCL10 abrogated NK cell recruitment, which established that CXCL10 mediates intrahepatic NK cell infiltration. Additionally, while infection of wild type mice resulted in caspase 3 cleavage at day 1, anti-AGM1 antibody treatment diminished the expressions of cleaved caspase 3 and TUNEL^+^ cells in the liver (Fig. 3B and C). Together, these results established a causal relationship between NK cell infiltration and liver cell death during early phase of DENV infection.

### Intrahepatic CD8^+^ T Cells are Cytotoxic against Infected Targets

**Figure 4:**
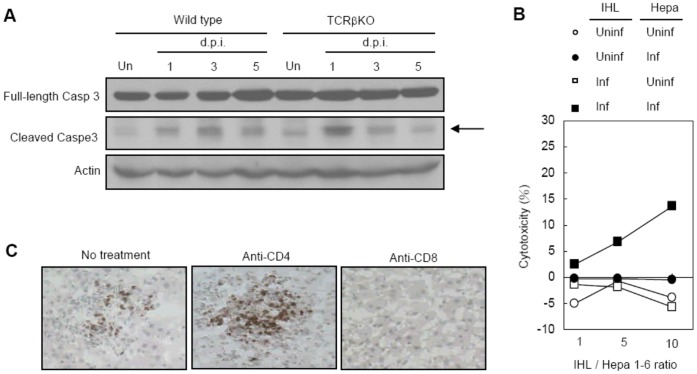
Intrahepatic T cells are cytotoxic against DENV-infected hepatocytes. (A) Liver lysates were collected from uninfected and infected wild type and TCRβ KO mice. Arrow points to cleaved capase 3. Data presented are representative of three independent experiments (*n = *3 per time point in each experiment). (B) Uninfected (Uninf) or infected (Inf) Hepa 1–6 were co-cultured with IHLs isolated from uninfected (Uninf) or infected (Inf) mice at different ratio. Data presented are representative of three independent experiments (*n = *3 per time point in each experiment). (C) Liver cryosections from infected mice at day 5 with or without anti-CD4 and anti-CD8 antibody were stained with TUNEL reagents. Data presented are representative of three independent experiments (*n = *3 per time point in each experiment).

Since caspase 3 cleavage was observed in liver not only in day 1 but also days 3 and 5 after infection (Fig. 1C) and T cell infiltration peaked at day 5 (Fig. 2D), we employed TCRβ KO mice to investigate whether T cells mediate liver cell death at later time points. While caspase 3 cleavage was detectable at days 1 and 3, it was almost completely diminished in TCRβ KO mice at day 5 after infection (Fig. 4A). These results strongly indicate that T cells are the cause of liver cell death at day 5 after infection. Results of *in vitro* assay showed that intrahepatic leukocytes from infected wild type mice were cytotoxic against DENV-infected Hepa 1–6 targets. At an effector-to-target ratio of 10∶1, the cytotoxicity was about 15% (Fig. 4B). Depletion of CD8^+^ but not CD4^+^ T cells completely eliminated TUNEL^+^ cells in the liver (Fig. 4C). Thus, intrahepatic CD8^+^ T cells and their cytotoxic activity against DENV-infected hepatocytes is the cause of liver cell death.

### Both Intrahepatic and Splenic CD8^+^ T Cells Recognize NS4B_99–107_ Epitope

**Figure 5:**
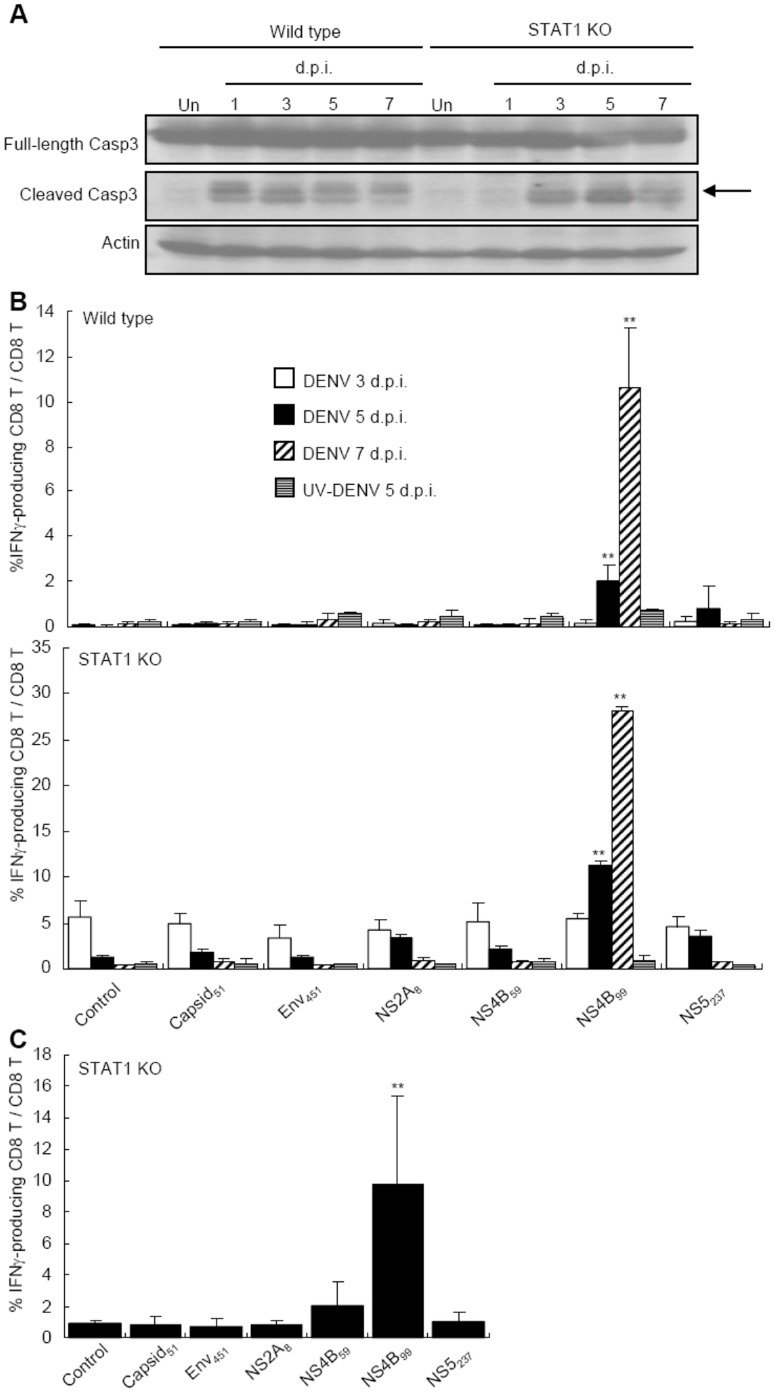
Intrahepatic as well as splenic CD8^+^ T cells recognize NS4B_99–107_ epitope. (A) Liver lysates were collected from uninfected and infected wild type and STAT1 KO mice. Arrow points to cleaved caspase 3. Data presented are representative of three independent experiment (*n = *3 per time point in each experiment). (B) Splenocytes were isolated from wild type and STAT1 KO mice at day 3 (empty), 5 (darkened), and 7 (hatched) after infection and at day 5 after UV-inactivated DENV injection (horizontal line). (C) IHLs were isolated from wild type and STAT1 KO mice at 5 days after DENV infection. Splenocytes and IHLs were stimulated with or without (control) indicated peptides. The percentages of IFNγ-producing CD8^+^ T cell within the total CD8^+^ T population were analyzed by flow cytometry. Data presented are representative of four independent experiments (*n = *3 per time point in each experiment). ** *P* value <0.01 compared with the control group.

Core_51–59_, NS2A_8–15_, NS4B_99–107_, and NS5_237–245_ were identified to be DENV-specific epitopes that are recognized by splenic CD8^+^ T cells in DENV clone S221-infected mice [21]. Since DENV 16681 share the same peptide sequence with the parental D2S10 virus in these positions, we asked whether the intrahepatic CD8^+^ T cells recognize any of these epitopes. Interestingly, infection of both wild type mice and STAT1 KO mice resulted in intrahepatic caspase 3 cleavage at days 3 and 5 though cleaved caspase 3 was not observed in STAT1 KO mice at day 1 due to their lack of functional NK cells [30] (Fig. 5A). Figure 5B shows that splenic CD8^+^ T cells in both DENV 16681-infected wild type and STAT1 KO mice recognized only NS4B_99–107_ but not other peptides. NS4B_99–107_-reactive CD8^+^ cells expanded in both wild type and KO mice from days 3 to 7 after infection. Injection of UV-inactivated DENV did not elicit CD8^+^ T cell response to any of the peptides tested (Fig. 5B). Taking advantage of the greater magnitude of CD8^+^ T cell response in STAT1 KO mice, we stimulated intrahepatic cell from infected STAT1 KO mice with different peptides. The results show that intrahepatic CD8^+^ T cells, like their splenic counterparts, recognized NS4B_99–107_ but not other peptides (Fig. 5C), indicating that intrahepatic CD8^+^ T cells that recognize NS4B_99–107_ constitute at least one of the major cytotoxic T cell populations that kill infected hepatic cells.

## Discussion

We have observed the correlations between T cell activation, hepatic cellular infiltration and elevated levels of AST and ALT in DENV-infected immunocompetent animals [21]. In this study, employing the same model we demonstrated that NK and T cells are recruited to the liver at different phases of infection. We provided direct evidence to show that while recruited NK cells are responsible for liver cell death at an early time point, intrahepatic infiltrating CD8^+^ T cells are for later time point. Infiltrating CD8^+^ T cells are cytotoxic against DENV-infected hepatocytes and NS4B_99–107_-reactive cells constitute at least one the major intrahepatic CD8^+^ T cell populations.

It is reported earlier that recombinant CXCL10 is able to inhibit the binding of DENV to heparan sulfate on hepatoma cell surface [31]. Here we showed that anti-CXCL10 antibody abolishes NK cell infiltration and that NK cell infiltration is critical to liver cell death at early phase of DENV infection. Thus, it appears that DENV-induced CXCL10 production is beneficial as well as harmful to the infected host. Through competition for heparan sulfate, CXCL10 inhibits DENV infection, but in the mean time, CXCL10-mediated recruitment of NK cells causes liver cell death.

*In vitro* studies show that DENV infection causes liver cell death. DENV infection of Hepa 1–6 induces cell death through a mitochondria-mediated pathway [32], while infection of HepG2 causes cell death through the upregulations of CD95 [33], CD137 [34] and TRAIL [35]. Studies showed that poly I:C-induced intrahepatic infiltrating NK cells express high levels of TRAIL and are responsible for liver damage [36]. Blocking TRAIL in EMCV-infected mice diminishes NK cell cytoxicity, which demonstrated that NK cell anti-EMCV response is TRAIL-mediated [37]. We showed in this study that DENV was transiently detected in the liver in the early phase of infection (Fig. 1C) during which time NK cells cause liver cell death (Fig. 3). It is our speculation that DENV infection of the liver, although very transient, up-regulates death receptors and renders hepatocytes susceptible to NK cell killing at early time point.

STAT1 KO mice are known to have impaired NK cell cytotoxic function. The mice are unable to reject NK sensitive tumor [30]. Our data showed that caspase 3 cleavage was induced only in the liver of infected-wild type but not -STAT1 KO mice at day 1 after infection. These results together with the anti-Asialo GM1 depletion experiment confirm the contribution of NK cells in DENV-induced liver injury during early phase of infection.

Yauch et al. observed specific anti-DENV CD8 T cell response in the spleen of mice infected by DENV S221 [29]. Although the sequences of capsid_51–59_ (K^b^), NS2A_8–15_ (K^b^), NS4B_99–107_ (D^b^_)_ and NS5_237–245_ (D^b^) peptides in DENV 16681 are identical to that in the parental D2S10, our study showed that splenic as well as intrahepatic CD8^+^ T cells from DENV 16681-infected mice recognize only NS4B_99–107_. In testing overlapping peptides Beaumier et al. found that CD8^+^ T cells in wild type mice infected with DENV NGC recognize NS4a (aa 249–265) and NS5 (aa 521–537) but not other peptides [38]. Since NS4a (aa 249–265, LLAIGCYSQVNPITLTA) contains the peptide sequence of NS4B_99–107_ (YSQVNPITL), our study and that of Beaumier et al. confirm that NS4B_99–107_ is the dominant CD8^+^ T cell epitope in H-2D^b^ mice.

It is interesting that immunization with pool peptides capsid_51–59_, NS2A_8–15_, NS4B_99–107_ and NS5_237–245_ induces CD8^+^ T cell protective immunity by lowering viral load. And, peptide (capsid_51–59_, NS2A_8–15_, NS4B_99–107_ and/or NS5_237–245_)-pulsed target cells were killed *in vivo* [29]. By the same assay, Beaumier et al. showed that DENV-immune mice rapidly clear NS4a (aa 249–265)- but not NS5 (aa 521–537)-pulsed targets [38]. In our study, both splenic and intrahepatic CD8^+^ T cells in the infected mice recognize NS4B_99–107_ epitope, and intrahepatic infiltrating CD8^+^ T cells are antigen-specific and not by-stander cells. While splenic CD8^+^ T cells are shown to be protective [29], our results showed that intrahepatic CD8^+^ T cells are cytotoxic and cause liver cell death. Therefore, CD8^+^ T cell immune response induced by DENV infection is a double-edge sword, on one hand it clears the virus yet when recruited to the liver they cause injury.

Elevated liver enzymes are reported in other hemorrhagic virus infections, such as Ebola and Crimena-Congo hemorrhagic fever [39], [40]. Apoptotic cells are found in the liver of yellow fever virus-infected patients [41]. Interferon-signaling deficient mice infected with Sindbis virus or Crimean-Congo hemorrhagic fever virus exhibit high liver enzymes and hepatomegaly [42], [43]. Infection of SCID mice with mouse-adapted Marburg hemorrhagic fever virus induces lethality and significantly elevated liver enzymes [44], [45]. Therefore, it appears that liver injury is common to infection by most hemorrhagic viruses. Although mouse differs from human in the composition of immune cells in the blood, the results of our study suggest that the involvement of NK and CD8^+^ T cells can not be ignored.

In summary, DENV can be detected in the serum and liver transiently after intravenous infection. DENV-triggered expression of CXCL10 recruits NK cells to the liver which cause liver cell death early after infection. Intrahepatic leukocytes recruited to the liver at later time points are cytotoxic against DENV-infected hepatocytes. The intrahepatic infiltrating CD8^+^ T cells are responsible for liver cell death and they recognize NS4B_99–107_. Our study indicate that NK and CD8^+^ T cells are both critical to dengue liver injury and that intrahepatic NS4B_99–107_-specific CD8^+^ T cells are the major cytotoxic T cell population that kill infected hepatic cells.
